# Estimating the duration of seropositivity of human seasonal coronaviruses using seroprevalence studies

**DOI:** 10.12688/wellcomeopenres.16701.3

**Published:** 2021-12-21

**Authors:** Eleanor M. Rees, Naomi R. Waterlow, Centre for the Mathematical Modelling of Infectious Diseases COVID-19 Working Group, Rachel Lowe, Adam J. Kucharski

**Affiliations:** 1 Centre for Mathematical Modelling of Infectious Diseases, London School of Hygiene & Tropical Medicine, London, UK; 2 Centre on Climate Change and Planetary Health, London School of Hygiene & Tropical Medicine, London, UK

**Keywords:** Seasonal coronavirus, Seroprevalence, Catalytic model, waning immunity

## Abstract

**Background:** The duration of immunity against severe acute respiratory syndrome coronavirus 2 (SARS-CoV-2) is still uncertain, but it is of key clinical and epidemiological importance. Seasonal human coronaviruses (HCoV) have been circulating for longer and, therefore, may offer insights into the long-term dynamics of reinfection for such viruses.

**Methods:** Combining historical seroprevalence data from five studies covering the four circulating HCoVs with an age-structured reverse catalytic model, we estimated the likely duration of seropositivity following seroconversion.

**Results:** We estimated that antibody persistence lasted between 0.9 (95% Credible interval: 0.6 - 1.6) and 3.8 (95% CrI: 2.0 - 7.4) years. Furthermore, we found the force of infection in older children and adults (those over 8.5 [95% CrI: 7.5 - 9.9] years) to be higher compared with young children in the majority of studies.

**Conclusions:** These estimates of endemic HCoV dynamics could provide an indication of the future long-term infection and reinfection patterns of SARS-CoV-2.

## Introduction

Severe acute respiratory syndrome coronavirus 2 (SARS-CoV-2), a novel beta coronavirus, was first detected in December 2019 and has since spread globally causing high morbidity and mortality. There is evidence of some short-term sterilising immunity (protection against reinfection and symptoms) following infection with SARS-CoV-2 ^1^, but also some reports of reinfection ^2^. However, there is currently limited evidence on the duration of immunity conferred by SARS-CoV-2 infection. Given the limited duration of SARS-CoV-2 circulation to date, the dynamics of antibody responses of seasonal human coronaviruses (HCoV) could provide insights into the possible long-term potential for reinfections ^3^. The duration of immunity following infection is of both clinical and epidemiological importance, as it provides information as to how long previously infected individuals may no longer be at risk of infection and disease, as well as influencing the long-term dynamics of epidemics ^4^ and enabling the interpretation of population-wide serological data ^5^.

There are four circulating HCoVs: HCoV-NL63 and HCoV-229E (alpha coronaviruses), HCoV-OC43 and HCoV-HKU1 (beta coronaviruses). HCoV-OC43 and HCoV-229E were first identified in the 1960s, but HCoV-NL63 and HCov-HKU1 were not identified until 2004 and 2005 respectively ^6, 7^. Like SARS-CoV-2, these typically cause respiratory tract infections. A small number of human challenge studies have looked at the duration of immunity to these viruses. Callow *et al.* ^8^ found that six out of nine participants were reinfected when challenged with HCoV-229E again one year later, as measured by a rise in IgG antibodies and viral shedding. However, the period of viral shedding was shorter following the second inoculation, and none of the participants developed symptoms. Reed ^9^ found that reinfection did not occur when participants were re-inoculated with a homologous strain approximately one year following infection, but participants had partial immunity against reinfection with a heterologous strain. Taken together these results suggest that immunity against infection with a homologous strain could last at least one year ^8, 9^.

There are also a small number of cohort and community-based surveillance studies which have looked at reinfection of seasonal HCoV. One study looked at HCoV reinfection in a small cohort of ten individuals over 35 years and found the median reinfection times to be 30 months, but with reinfection often occurring at 12 months ^10^. A larger study looking at data from Flu Watch, a community cohort study which measures the incidence and transmission of respiratory viruses, found that between 2006 and 2011, eight subjects were reinfected with a seasonal HCoV (of 216 with confirmed first infection), and the time between reinfection ranged from 7 to 56 weeks. None of these reinfections were with the same strain, providing some evidence of lasting immunity ^11^. However, a community surveillance study of 483 participants conducted in Kenya in 2010 over six months found evidence of high numbers of repeat infections of HCoV-NL63 (20.9%), HCoV-OC43 (5.7%), and HCoV-229E (4.0%). The majority of these reinfections showed reduced virus replication in the second infection, and a lower proportion of individuals had symptoms following the second infection ^12^. Furthermore, another study conducted in New York City which included 191 participants found that reinfections with the same strain can occur within one year ^13^. Care should be taken with the interpretation of these studies since we do not know the background exposure rates, and this will influence the estimates of duration of immunity.

If infections are fully immunising – as is the case for pathogens like measles and varicella zoster – then seroprevalence would be expected to accumulate over time ^14^, and hence with age, with little waning of responses. The dynamics can therefore be captured with catalytic models of seroconversion ^15^, which enables estimation of the force of infection (FOI, the rate at which susceptible individuals acquire infection and seroconvert). In contrast, when individuals serorevert, i.e. their immunity wanes by the progressive loss of protective antibodies against a disease over time, ‘reverse catalytic models’ can jointly estimate FOI and waning of immunity ^16^. Variation in FOI with age may further complicate the dynamics, particularly if a high infection rate in children is followed by a lower rate in adults as well as waning of seroprevalence. To understand how seroconversion, waning and age-variation in infection risk could shape population-level seroprevalence, we combine age-stratified data with age-structured reverse catalytic models, and estimate the likely duration of seropositivity following seroconversion for the four seasonal coronaviruses.

## Methods

Human seroprevalence from four different human coronavirus strains (229E, HKU1, NL63, and OC43) were identified in a recent systematic review ^7^. Studies which did not include estimates for individuals under 10 years old ^17^ were excluded, as well as studies with which only reported two age groups ^18^. A total of six different studies were included, covering the four seasonal HCoVs, with some studies reporting on multiple strains ^19– 24^. Two studies were reported separately for two different strains, but the overall study population was the same ^21, 22^. A summary of these studies is presented in Table 1. The different assays used in each study for the different strains is shown, and where the antibody detected was specified this is included in the table. To account for maternal immunity individuals aged ≤1 year were excluded. The full dataset used for this analysis can be found as underlying data ^25^.

**Table 1.** Characteristics of studies used to fit the model.

Strain	Author (year published)	Pubmed ID	Sample size	Country/ region	Years sampled	Assay	Antigen	Assay cut-off
HCoV-HKU1	Chan (2009)	19342289	709	Hong Kong	Not specified	ELISA (IgG)	S protein	Mean + 3SD (OD>0.495)
	Zhou (2013) ^a^	24040960	789	China	1999 – 2011	IFA (IgG)	S protein	>1:20
HCoV-OC43	Zhou (2013) ^a^	24040960	789	China	1999 – 2011	IFA (IgG)	S protein	>1:20
	Monto (1974) ^c^	4816305	910	USA	1965 – 1969	CF or HI	Whole virus	<1:8 to >1:8 or 4-fold rise
	Sarateanu (1980)	6248465	3,016	Germany	1974 – 1976	HI	Whole virus	>1:8
HCoV-NL63	Shao (2007) ^b^	17889596	243	USA	2003 – 2004	ELISA (IgG)	N protein	OD>0.2 at dilution of 1:80 or greater
	Zhou (2013) ^a^	24040960	789	China	1999 – 2011	IFA (IgG)	S protein	>1:20
HCoV-229E	Shao (2007) ^b^	17889596	243	USA	2003 – 2004	ELISA (IgG)	N protein	OD>0.2 at dilution of 1:80 or greater
	Zhou (2013) ^a^	24040960	789	China	1999 – 2011	IFA (IgG)	S protein	>1:20
	Cavallaro (1970) ^c^	5504709	307	USA	1966	Neutralization	Whole virus	>1:4

Human coronavirus (HCoV), Enzyme-linked immunosorbent assays (ELISA), immunofluorescence assays (IFA), complement fixation (CF), hemagglutination inhibition assays (HI), Immunoglobulin G (IgG), standard deviation (SD), optical density (OD). Studies which occurred in the same setting are denoted by the superscripts, a, b and c.

To explore the duration of antibody persistence for different seasonal coronaviruses, where detectable antibodies is defined as seropositivity, we developed age-structured reverse catalytic models. The basic reverse catalytic model follows individuals from birth and assumes that there is a constant FOI (λ), which is independent of age (a) and calendar year, and that immunity (as measured by serological status) wanes over time, at a rate ω. This model also assumes that the mortality rate for susceptible and infectious individuals is the same. The expression for the proportion of individuals age *a* who are seropositive, *z(a)*, in the reverse catalytic model is as follows:

$$z(a)=\frac{\lambda }{\lambda +\omega }(1-e^{-a(\lambda +\omega )})$$

where λ is the FOI, ω is seropositivity waning rate and *a* is age. The duration of antibody persistence was estimated as follows:

*Duration of antibody persistence* = 1/ω

We then extended the reverse catalytic model to allow for a different FOI by age. The expressions for seroprevalence in the reverse catalytic model with age-varying FOI are as follows:

$$\begin{array}{l} z(a)=\frac{\lambda_{1}}{\lambda_{1}+\omega }(1-e^{-a(\lambda_{1}+\omega )})\;when\;a<a_{0}\\ z(a)=(\frac{\lambda_{1}}{\lambda_{1}+\omega }(1-e^{-a_{0}(\lambda_{1}+\omega )})\;-\;\frac{\lambda_{2}}{\lambda_{2}+\omega })\;(e^{-(\lambda_{2}+\omega )(a-a_{0})}\;)+\;\frac{\lambda_{2}}{\lambda_{2}+\omega }\;when\;a\geq a_{0}\\ \lambda_{2}=\lambda_{1}\;\alpha \end{array}$$

Where z(a) is those who are seropositive at age a, λ₁ is the FOI in young age groups, λ₂ is FOI in the old age group, ω is waning, *a* is age, *a _0_* is the age cut-off used to define the young and old group, and the relative change in FOI, α, is the change in FOI in the older age group. In our analysis, we allowed λ _1_ to vary by study and strain, to account for local differences in population-level transmission dynamics, while the average rate of waning within a given individual was assumed to be universal and was jointly estimated across all studies and strains. This means that one overall estimate of waning was obtained. Some of the studies occurred in the same setting, and so the underlying contact patterns were presumed to be the same (in total we identified five settings). Therefore, the relative change in FOI (α) and the age at cut-off ( *a _0_*) were jointly estimated across settings. This model assumes no cross-protection between strains. Annual attack rates were calculated after estimating the FOI using the following expression,

$$Attack\;rate=1-e^{-\lambda }.$$

To reflect uncertainty in current knowledge about the transmission dynamics of HCoVs, weakly informative distributions were chosen as priors for ω, the rate of waning over time. Specifically uniform priors from 0 to 5 years. For the FOI, there is little information on the attack rate of HCoVs. However, there have been several systematic reviews and meta-analyses looking at influenza in unvaccinated individuals which have reported the attack rates to range between 15.2% – 22.5% in children and 3.5% – 10.7% in adults ^26– 28^. Modelling studies using serological influenza data predicted estimates from 20 – 60% ^29, 30^. Based on the epidemiology of these viruses in children ^31^, we expect the attack rate for HCoV may be lower. Therefore, we selected a Gamma distribution, with a mean of 0.3 (shape = 1.2 and scale = 0.25) and this corresponds to an attack rate of 26% and covers a range of plausible values. For the age at cut-off (a *_0_*), uniform priors from 0 to 20 years were chosen as we were interested in the difference in FOI in children and young adults. For the relative change in FOI (α) we did not have any prior information. Therefore, we selected a prior with median 1, which presumes no difference between FOI in young compared with FOI in old and allowed for a range of plausible values using a gamma distribution (shape = 5, scale = 0.2).

Several sensitivity analyses were conducted to assess the robustness of these results. First, the choice of priors for the FOI was explored, and a less informed prior was tested (FOI ~ Normal (mean 0.3, standard deviation 0.5)). Second, waning was estimated by strain, instead of being jointly fitted across all studies. The relative change in FOI (α) and age at cut-off ( *a _0_*) were then held across all studies (instead of allowing them to vary by setting) to explore the impact on the estimate for waning. The impact of excluding the youngest age groups (≤1 year) was also explored, and a model was run which included individuals ≤1 year. The impact of the assay used in the study on the estimate of waning was also explored, where FOI was allowed to vary by study, alpha and the age at cut-off varied by setting and waning varied by assay (ELISA, IFA, HI and neutralisation). Finally, the primary model (age-varying FOI model) was fitted using only half the data (seroprevalence studies from two strains), to explore whether the results from one study was heavily influencing the results. For this model, waning, the relative change in FOI (α) and age at cut-off ( *a _0_*) were held across all studies. A description of these models is presented in Table 2.

**Table 2.** Description of models explored.

Model	Priors	Number of parameters
Main model: Reverse catalytic model with age-varying FOI (alpha and cut-off varying across settings) - More informed priors	FOI ~ gamma(shape = 1.2, scale = 0.25) Waning ~ uniform(0,5) Alpha ~ gamma(shape = 5, scale = 0.2) Cut-off ~ uniform(0,20)	21
Reverse catalytic model with age-varying FOI (alpha and cut-off varying across settings) - less informed priors	FOI ~ normal(0.3,0.5) Waning ~ uniform(0,5) Alpha ~ gamma(shape = 5, scale = 0.2) Cut-off ~ uniform(0,20)	21
Reverse catalytic model with age-varying FOI (alpha and cut-off varying across settings, waning varying by strain)	FOI ~ gamma(shape = 1.2, scale = 0.25) Waning ~ uniform(0,5) Alpha ~ gamma(shape = 5, scale = 0.2) Cut-off ~ uniform(0,20)	24
Reverse catalytic model with age-varying FOI (alpha and cut-off held across settings)	FOI ~ gamma(shape = 1.2, scale = 0.25) Waning ~ uniform(0,5) Alpha ~ gamma(shape = 5, scale = 0.2) Cut-off ~ uniform(0,20)	13
Reverse catalytic model with age-varying FOI (alpha and cut-off varying across settings) including data <1 Year	FOI ~ gamma(shape = 1.2, scale = 0.25) Waning ~ uniform(0,5) Alpha ~ gamma(shape = 5, scale = 0.2) Cut-off ~ uniform(0,20)	21
Reverse catalytic model with age-varying FOI (alpha and cut-off varying across settings, waning varying by assay)	FOI ~ gamma(shape = 1.2, scale = 0.25) Waning ~ uniform(0,5) Alpha ~ gamma(shape = 5, scale = 0.2) Cut-off ~ uniform(0,20)	24
Reverse catalytic model	FOI ~ gamma(shape = 1.2, scale = 0.25) Waning ~ uniform(0,5)	11

Force of infection (FOI), relative change in FOI (Alpha,α), age at which the FOI changes (Cut-off).

Bayesian inference was used to fit the sero-catalytic models to the seroprevalence data, using Markov chain Monte Carlo (MCMC) with the Gibbs sampling algorithm to estimate model parameters. To do so, we used the following binomial likelihood representing seropositivity by age (a), study (i) and strain (j)

$$y_{ija}\sim Binomial\;(P_{ija},N_{ija}),$$

where *N _ija_* is total number of individuals by age group, strain and study, and *P _ija_* is the proportion of individuals who are seropositive. The inference was implemented in RJags (version 4–10) ^32^. The Gelman-Rubin statistic was used to evaluate MCMC convergence, and a threshold of <1.1 was chosen. The effective sample size (ESS), which is the estimated number of independent samples accounting for autocorrelations generated by the MCMC run, was checked, and an ESS >200 was used. All analysis and calculations were performed using R version 3.6.1. Model selection was based on the lowest value of the widely applicable information criterion (WAIC) and the leave-one-out cross validation (LOO) using Pareto-smoothed importance sampling ^33, 34^. WAIC and LOO were estimated using the R package Loo (version 2.4.1) ^34^. All code is available here at GitHub ^25^.

## Results

**Figure 1.**
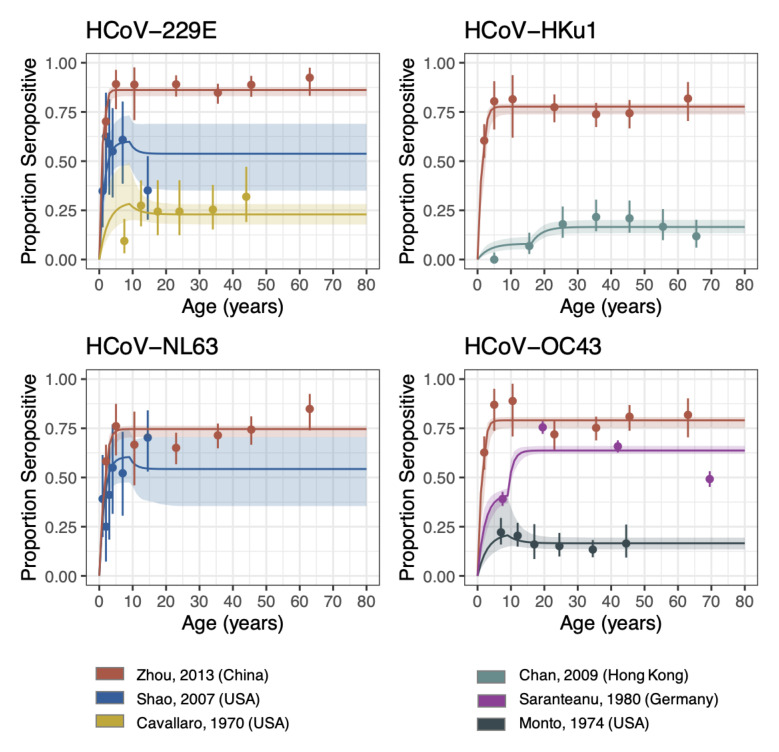
Reverse catalytic model with age-varying FOI. The points are the observed proportion of seropositive individuals from each study (with confidence intervals), i.e. the data that was fit to. The lines are the seroprevalence curves, sampled from the fitted model, where the shaded region represents the 95% credible interval of the predictive posterior distribution. FOI was allowed to vary by study, whilst the relative change in FOI (Alpha) and cut-off were allowed to vary by setting. Waning was jointly fit across all studies and strains.

**Figure 2.**
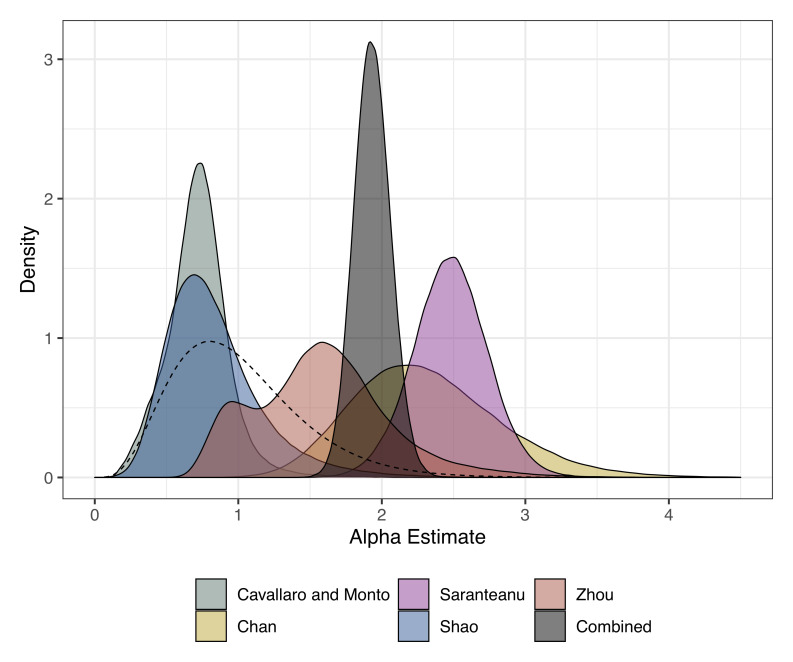
Posterior estimates for the relative change in FOI (alpha) from the age-varying reverse catalytic model for each study setting. The alpha estimate from the model where alpha and cut-off were simultaneously estimated across studies is shown in grey as “combined”. The prior is shown as a dashed line.

Using a reverse catalytic model, which allowed the FOI to change in individuals by age, we estimated the duration of antibody persistence for the four seasonal HCoVs. Despite having only four parameters by study, our model could capture the overall trends in most studies ( Figure 1). Waning was jointly fitted across all studies and strains to obtain one overall estimate, and the duration of antibody persistence was estimated to be 3.75 (95% credible interval [CrI]: 1.96 – 7.38) years ( Table 3). The FOI across all studies and strains in the young age group ranged from 0.02 (95% CrI: 0.01 – 0.05) to 1.06 (95% CrI: 0.57 – 1.68). The cut-off (age at which the FOI changes) ranged between 2.35 (95% CrI: 0.31 – 17.51) to 16.58 (95% CrI: 7.71 – 19.81) years. The relative change in FOI (Alpha) which measures the relative value of FOI in the young age group compared with the older age group ranged from 0.72 (95% CrI: 0.3 – 1.17) to 2.48 (95% CrI: 1.96 – 2.99). For three of the study settings, the FOI in the older age group was higher ( Figure 2). A sensitivity analysis was conducted using less informative priors for the FOI parameters, where a normal distribution was used (extended data Figure 1, Table 1 ^35^). This model estimated a shorter duration of antibody persistence [0.93 (95% CrI: 0.60 – 1.64) years]. The FOI across all studies and strains were higher, ranging from 0.09 (95% CrI: 0.04 - 0.16) to 3.22 (95% CrI: 1.95 - 4.85), with six studies reporting FOI estimates > one, which is equivalent to an attack rate of >63%. The relative change in FOI and cut-off were similar for both models. This model had a lower WAIC (536.4 compared with 545.9) and LOO (546.0 compared with 557.8), which suggests that this model may have an improved fit compared with the model with more informed priors, however, large standard errors (SE) were reported for both WAIC and LOO. Furthermore, the high FOI estimates indicate that this model may be less plausible ( Table 4). As an additional sensitivity we allowed the waning estimate to vary by strain (extended data Table 2 ^35^). This model estimated the duration of antibody persistence to be similar for all strains, ranging from 2.26 (1.06 – 5.07) years for HCoV-OC43 to 4.09 (1.91 – 9.60) years for HCoV-229E.

**Table 3.** Parameter estimates from the age-varying FOI reverse catalytic model (median [95% CrI]). FOI was allowed to vary across study, while waning was simultaneously estimated across all studies. The relative change in FOI (Alpha) and the cut-off were allowed to vary across study settings.

Strain	First Author	FOI (youngest age group)	Relative change in FOI (Alpha)	Age at which the FOI changes (cut-off)	Waning
HCoV-229E	Shao	0.40 (0.26 – 0.64)	0.78 (0.35 – 1.68)	9.5 (0.59 – 19.47)	0.27 (0.14 - 0.51)
	Zhou	1.06 (0.57 – 1.68)	1.57 (0.8 – 2.65)	2.35 (0.31 – 17.51)	
	Cavallaro	0.11 (0.06 – 0.3)	0.72 (0.3 - 1.17)	9.14 (0.57 - 19.28)	
HCoV-HKU1	Chan	0.02 (0.01 - 0.05)	2.27 (1.44 – 3.45)	16.58 (7.71 – 19.81)	
	Zhou	0.59 (0.32 – 0.89)	1.57 (0.8 – 2.65)	2.35 (0.31 – 17.51)	
HCoV-OC43	Zhou	0.64 (0.35 – 0.96)	1.57 (0.8 – 2.65)	2.35 (0.31 – 17.51)	
	Monto	0.07 (0.04 – 0.19)	0.72 (0.3 – 1.17)	9.14 (0.57 – 19.28)	
	Sarateanu	0.19 (0.11 – 0.35)	2.48 (1.96 – 2.99)	9.93 (7.34 – 14.84)	
HCoV-NL63	Zhou	0.50 (0.27 – 0.74)	1.57 (0.8 – 2.65)	2.35 (0.31 – 17.51)	
	Shao	0.41 (0.26 – 0.67)	0.78 (0.35 – 1.68)	9.5 (0.59 – 19.47)	

Human coronavirus (HCoV), force of infection (FOI).

**Table 4.** Comparison of duration of antibody persistence estimates from the different models explored.

Model	Reverse catalytic model with age-varying FOI (alpha and cut-off varying across settings) - More informed priors	Reverse catalytic model with age-varying FOI (alpha and cut-off varying across settings) - less informed priors	Reverse catalytic model with age-varying FOI (alpha and cut-off held across settings)	Reverse catalytic model
Duration of antibody persistence (years)	3.75 (95% CrI: 1.96 – 7.38)	0.93 (95% CrI: 0.60 – 1.64)	2.20 (95% CrI: 1.57 – 3.08)	7.69 (95% CrI: 6.25 – 9.09)
WAIC	545.9 (SE: 100.2)	536.4 (SE: 99.6)	622.1 (SE: 103.3)	717.2 (SE: 156.8)
LOO	557.8 (SE: 102.3)	546.0 (SE: 100.6)	632.5 (SE: 105.6)	718.5 (SE: 151.8)

Force of infection (FOI), relative change in FOI (Alpha), age at which the FOI changes (Cut-off), widely applicable information criterion (WAIC), leave-one-out cross validation (LOO), Standard Error (SE).

When the relative change in FOI and cut-off parameters were simultaneously estimated by setting (extended data Figure 2, Table 3 ^35^) the duration of antibody persistence was estimated to be shorter, 2.20 (95% CrI: 1.57 - 3.08) years, although the confidence intervals overlap with the main model. The FOI ranged from 0.04 (95% CrI: 0.03 - 0.06) to 0.88 (95% CrI: 0.67 - 1.19). The overall model WAIC (622.1 compared with 545.9) and LOO (632.5 compared with 557.8) were higher, indicating that this model did not have as much support, although the SEs reported were large for WAIC and LOO ( Table 4).

**Figure 3.**
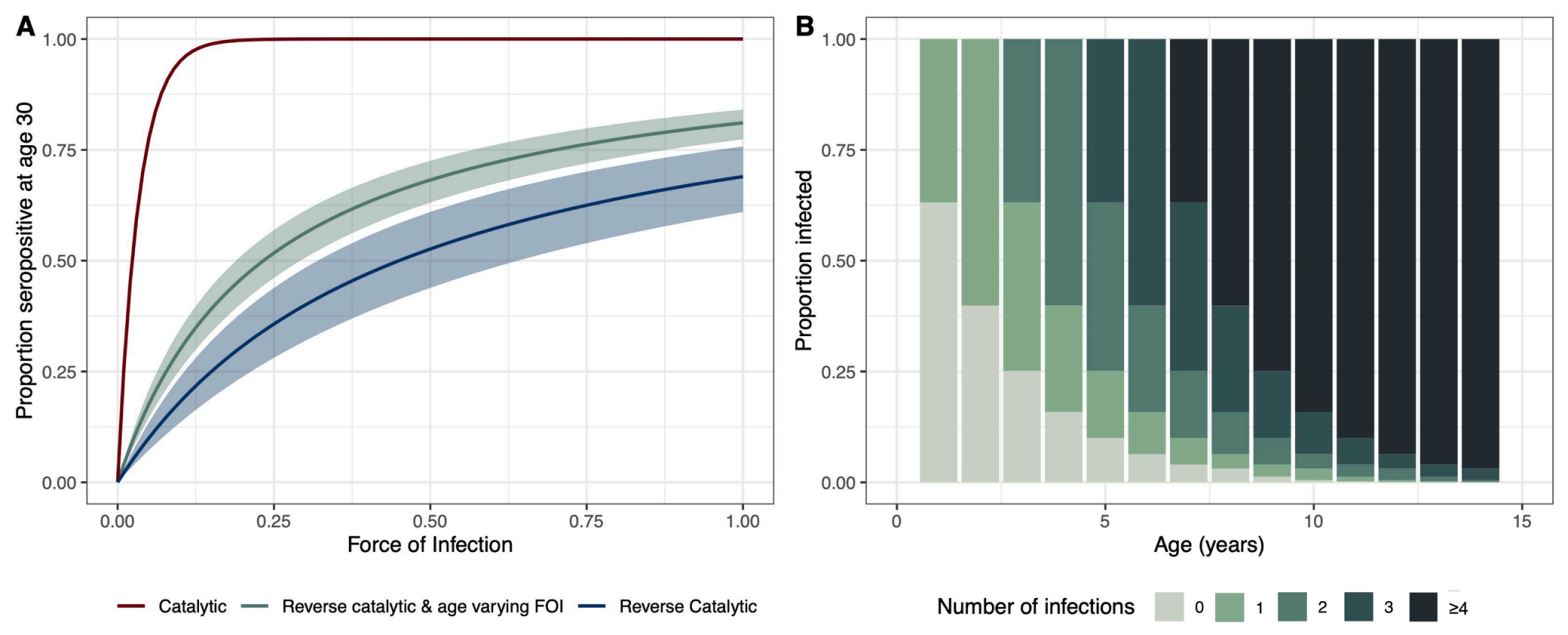
( **A**) Proportion of individuals age 30 who are seropositive for different estimates of force of infection (FOI). The catalytic model is shown in red, the reverse catalytic model in green, and the reverse catalytic model with age-varying FOI is shown in blue. Model estimates were used for the parameter values (relative change in FOI (alpha),1.93 [1.69 – 2.19]; waning, 0.45 [0.32 – 0.64]; cut-off, 8.49 [7.52 – 9.94]). ( **B**) Estimated proportion of individuals experiencing infections by age estimated from the age-varying reverse catalytic model (more informed priors) using the pooled median estimate across studies for FOI (0.46), and median estimates for waning (0.45), alpha (1.93) and cut-off (8.49).

We also tested a basic reverse catalytic model, where the FOI was not allowed to vary by age, and this model estimated a longer duration of antibody persistence (7.69 [95% CrI: 6.25 - 9.09] years; extended data Table 4, Figure 3 ^35^). The WAIC (717.2) and LOO (718.5) values for the basic reverse catalytic model were higher compared with the other models, indicating that this basic model did not have strong support among the models considered ( Table 4).

To explore the effect of excluding the youngest ages (≤1 year), a sensitivity analysis was done where these individuals were included within the analysis. The duration of antibody persistence was found to be slightly shorter (2.04 [95% CrI: 0.1.28 -1.4.76] years) and the FOI was found to be higher for all studies, ranging from 0.04 (95% CrI: 0.02 - 0.07) to 2.92 (95% CrI: 2.08 - 4.01); extended data Table 5, Figure 4 ^35^). The estimates for the relative change in FOI were found to be very similar to the model which excluded this age group.

As an additional sensitivity analysis, we refit the models using data for only two strains at a time, and estimated the FOI, waning and the relative change in FOI (extended data Table 6 ^35^). We found that although the results varied, the overall trends were the same, indicating that the model did not rely heavily on one dataset. The duration of antibody persistence varied from 1.80 years (95% CrI: 1.17 - 2.67) to 5.26 years (95% CrI: 2.53 - 13.56).

Finally, we explored the impact of the different assays used in the studies on the waning estimates. We allowed the waning estimate to vary by assay (extended data Table 7, Figure 5 ^35^), whilst allowing FOI to vary by study, and alpha and cut-off to vary by setting. This model estimated the duration of antibody persistence to be similar for ELISA (2.63 [95% CrI: 0.94-9.09] years), HI (1.08 [95% CrI: 0.44-3.33] years) and neutralisation (1.28 [95% CrI: 0.25-50.0] years) assays, but longer for IFA (7.69 [95% CrI: 3.03-14.29] years). The credible intervals were wide, likely due to the small number of studies by assay.

To demonstrate the relationship between FOI and seropositivity at age 30, we created simulated scenarios under different sero-catalytic models. Using the parameters for the relative change in FOI and waning estimated from the age-varying reverse catalytic model (where the relative change in FOI and the age at cut-off were simultaneously estimated across settings), we simulated the proportion of individuals aged 30 years that would be seropositive using a range of FOI estimates to show how the proportion changes using the different models. The catalytic model, which does not allow for seroreversion, results in the highest estimates of seropositivity at age 30 with increasing FOI. The age-varying FOI model results in higher estimates of seropositivity at age 30 compared with the reverse catalytic model. This is due to the FOI which was estimated to be almost twice as high in the older age group (with age at cut-off 8.49 [7.52 – 9.94] years) in the age-varying FOI model ( Figure 3A). We further explored the relationship between FOI, attack rates and the estimated number of infections by age. We used the pooled estimate across all studies of FOI to estimate the proportion exposed at a given age to provide an indication of how many infections we might expect to see by age under our modelling assumptions ( Figure 3B). We estimate that by two years, over 50% of the population will have at least one infection, and by age ten over 75% will have had more than four infections.

## Discussion

To date, there has been limited evidence about the duration of immunity to SARS-CoV-2. Given the inevitable right censoring of data during an emerging infectious disease pandemic, understanding the duration of protection following infection with HCoV could help provide insights which will be relevant to SARS-CoV-2. Using an age-varying reverse catalytic model, we estimated the overall duration of immunity, as measured by seropositivity, to be between 0.9 (95% CrI: 0.6 - 1.6) years and 3.8 (95% CrI: 2.0 - 7.4) years for HCoV’s. When waning was estimated by strain, we found comparable estimates of the duration of seropositivity, indicating that the assumption that waning is similar across strains holds true. Previous studies have produced varied estimates for the duration of immunity for HCoVs. One study estimated the median duration of immunity to be 2.5 years ^10^, and Reed found immunity lasts at least one year ^9^. However, several studies have reported reinfection occurring in less than one year ^8, 11– 13^. Aldridge *et al*. ^11^ found that reinfection with HCoV did not occur with the same strain, but Kiyuka *et al*. ^12^ found reinfection frequently occurred with the same strain within a six month period. The reverse catalytic model assumes that waning occurs at a constant rate, however, individuals may become reinfected within a shorter time period than average, and conversely some will take longer. Some evidence also exists for the duration of immunity to SARS-CoV-2. A recent survey of health care workers in Oxford, UK, found that protection against reinfection with SARS-CoV-2 lasts at least six months ^36^, whilst another study of health care workers from across the UK conducted by Public Health England found that immunity lasts for at least five months ^2^. This seems to align with what is known about reinfection in seasonal HCoVs. However, these studies only followed up individuals for six months and five months respectively, and longer follow-up times are needed. Future studies could also work to untangle the relationship between seroreversion as a result of waning homotypic antibody responses and antigenic evolution leading to a mismatch between prior immunity and circulating viruses ^37^.

More informed priors for the FOI based on attack rates for influenza, resulted in higher estimates for the duration of seropositivity. When we used less informed priors for the FOI, a lower estimate of duration of seropositivity was obtained. However, this model produced higher estimates of FOI, with six studies reported FOI estimates in the young age group greater than one (attack rate >63%). There is limited information on the attack rate of seasonal HCoV, however there have been numerous studies looking at influenza. Previous systematic reviews have estimated the attack rate of influenza to be between 3.5% and 22.5% ^26– 28^, whilst modelling studies have estimated this to be higher, 20 – 60% ^29, 30^. Based on reporting rates of seasonal HCoV we would expect the attack rate to be lower than influenza. Therefore, this suggests that the results from the model with less informative priors are less plausible. Maternally derived immunity may also have a role, protecting young infants from infection ^38^. We tested this with a model which included individuals ≤1 year. This resulted in a shorter estimate of the duration of antibody persistence, and a higher FOI, suggesting that maternal immunity may be important.

A wide range of different assays were used in the studies we considered in our analysis, including enzyme-linked immunosorbent assays (ELISA), immunofluorescence assays (IFA), western blots, and complement fixation (CF), hemagglutination inhibition assays (HAI) and neutralisation assays. Neutralisation assays are considered to be the gold standard as they measure the ability of the sera to inhibit viral processes ^7, 39^. Only Cavallaro and Monto ^22^ used a neutralisation assay. Other assays, such as ELISA and IFA, do not assess the functionality of the antigen, but instead detect the presence of antibodies in a sample. Zhou *et al*. ^19^ used IFA to detect levels of IgG antibodies. When we allowed the waning estimate to vary by assay, we found a similar estimates of antibody persistence for ELISA, HI and neutralisation assays, ranging from 1.1 years to 2.6 years, and these are comparable to the estimates from the main model. However, for IFA, we observed a longer estimate of 7.7 years (CrI: 3.0-14.3). Due to the small number of studies, the credible intervals were large, particularly for the IFA and neutralisation assay, which only had one study setting for each assay. This highlights the need for more studies, and better standardisation of assays. A recent study provided evidence that IgG antibodies in SARS-CoV-2 are correlated with neutralising antibodies, and may therefore act as a correlate of sterilising immunity ^40^, whilst another study suggested that neutralizing antibodies may be correlated with protection against reinfection ^1^. Therefore, although antibody prevalence does not equate to immunity for seasonal HCoVs, prevalence of IgG antibody may be a good correlate of immunity. However, all of these assays only assess humoural immunity, and it is thought that cellular immunity also has a role SARS-CoV-2, and so it is likely to be also important in seasonal HCoVs ^41– 43^.

The seroprevalence surveys included in this study were conducted in different countries and settings (USA, China, Germany and Hong Kong), as well as in different time-periods (ranging from 1965–2011). It is likely that there are differences in social structure and contact patterns between these settings. Furthermore, individual level data was not available for these studies, and instead aggregated data was used. Finer resolution, particularly for the younger age groups, would have helped to provide more certainty with these estimates. In addition, we did not take into consideration cross-protection between seasonal coronavirus strains. There is some evidence of cross protective immunity between seasonal coronavirus strains, and in settings where there is co-circulating HCoV strains, this may lead to a higher prevalence. There is also evidence that there is cross-reactivity between different coronaviruses, which may lead to false positive results. A recent systematic review found that there was some cross-reactivity that occurred within alpha (HCoV-229E and HCoV-NL63) and beta (HCoV-OC43 and HCoV-HKU1) coronaviruses, but minimal reactivity between alpha and beta coronaviruses ^7^. However, it is not clear whether cross-reactivity equates to cross-protection. False positives due to cross-reactivity would lead to an over-estimation of seroprevalence in a setting. This would lead to a higher plateau in older ages, and therefore generally lead to an over-estimation of both the FOI and the duration of antibody persistence. We also did not account for seasonality within this model, which may have under-estimated our FOI. Ferrari *et al.* ^44^ found that ignoring seasonality may overemphasize the role of adults in the transmission, however, this was observed in measles in Niger, with outbreak peaks ranging over several orders of magnitude, and long periods between epidemics. The epidemic profile is different for seasonal coronaviruses, and therefore, this is unlikely to apply in this context. Whitaker & Farrington ^45^ found that accounting for seasonality resulting from past epidemics only had a marginal effect on the estimates, and that regular epidemic dynamics do not strongly bias the catalytic model. The time of year data collection occurred may influence seropositivity estimates, particularly given that the duration of antibody persistence is estimated to range between 0.9 (95% CrI: 0.6 - 1.6) years and 3.8 (95% CrI: 2.0 - 7.4) years. Data collection during high transmission periods would lead to an overestimate of both the FOI and the duration of antibody persistence. All the studies (except for Chan *et al.* ^23^ who did not report this information), included within this analysis collected data over at least a six-month period. For this reason, the timing of data collection is unlikely to have biased our results. We also assume an overall FOI by age, and we do not account for differences in population susceptibility, for example health care workers or immunocompromised individuals. Despite these limitations, the duration of immunity estimated in this study is in line with literature estimates, suggesting the age-varying reverse catalytic model was able to capture overall dynamics.

Numerous studies have looked at the age pattern of HCoV patients presenting to hospital and healthcare settings, and predominantly found that the burden of disease is higher in younger children and the elderly ^46– 48^. However, it is likely that these age groups may have more severe symptoms and are therefore more likely to be reported. In contrast, seroprevalence data makes it possible to examine the whole population for evidence of past exposure, and hence can provide a clearer understanding of the underlying transmission dynamics of disease, rather than just the resulting burden.

In this study, when the relative change in FOI and the age of cut-off were simultaneously estimated across studies, we found that the FOI was estimated to be twice as high in the older age group (in this case, those over 8.49 [CrI: 7.52 - 9.94] years), compared with the younger age group. A similar pattern was observed for three of five settings when the relative change in FOI and the cut-off age were allowed to vary by setting. This suggests that older children and adults may be important for the transmission of seasonal HCoVs in some settings. A previous study looking at social mixing patterns in Europe ^49^ found that children are expected to have the highest incidence during the initial stages of an epidemic as a result of their social mixing patterns, and this is what is found for some diseases, such as seasonal influenza, where there is evidence young children drive transmission ^50, 51^. However, a more recent study looking at a large scale dataset of movement and contact patterns in the United Kingdom data found contact intensity was highest in the 18–30 year age group when looking at all types of contacts (conversational, which was defined as face-to-face conversation of three or more words, and physical), although for physical alone, those aged 5–9 years had the highest contact ^52^. Therefore, any association between contact intensity and transmission will depend on the contacts considered, particularly if a pathogen is more commonly spread via conversational contacts or via prolonged physical contacts. One possible explanation for the higher FOI we estimate in older age groups is that conversational contacts – which are typically higher in volume but lower in duration and intensity – could be more important for the transmission of seasonal HCoVs.

The results from this study are in accordance with what studies have observed in children during the coronavirus disease 2019 (COVID-19) pandemic, with low numbers of cases reported in young age groups, and several large seroprevalence studies have reported lower seroprevalence in children compared with adults ^53, 54^. As well as differences in contact structure, this could be explained in part by reduced susceptibility to acquisition of infection; a meta-analysis of contact tracing studies found that children had 56% (31% – 71%) lower odds of becoming an infected contact compared with adults ^55^.

The duration of immunity to SARS-CoV-2 is still largely unknown and is of significance for the interpretation of population wide serological data, the understanding of the long-term dynamics of the epidemic, as well as of clinical importance. Given the long-term circulation of seasonal HCoVs, data on these related coronaviruses could provide indications of the possible future dynamics of SARS-CoV-2. With infection likely to become endemic in parts of the world, the duration of antibody-mediated immune responses will be particularly important in shaping transmission patterns in years to come. Using seroprevalence data, in this study we estimated the duration of seropositivity to seasonal HCoVs following seroconversion to be between 0.9 (95% CrI: 0.6 - 1.6) years and 3.8 (95% CrI: 2.0 - 7.4) years. We allowed the FOI to vary by age group and found it to be lower in young children (≤8.5 years) compared with older children and adults, which is corroborated with what has been observed in the COVID-19 pandemic. This suggests individuals in settings with endemic HCoVs accumulate multiple infections over the course of their lifetime, punctuated by periods of waning seropositivity against circulating viruses.

## Data availability statement

### Underlying data

Zenodo: erees/seasonalHCoV: First release. https://doi.org/10.5281/zenodo.5707764 ^25^

This project contains the following underlying data:

- Data extracted from Huang *et al.* ^7^ (“ 41467_2020_18450_MOESM7_ESM-1.csv”)

GNU General Public License v3.0.

### Extended data

Zenodo: Extended data: Estimating the duration of seropositivity of human seasonal coronaviruses using seroprevalence studies. https://doi.org/10.5281/zenodo.5784018 ^35^.

This project contains the following extended data

• SupplementaryMaterial.pdf
  • Sensitivity analysis: Less informed priors for FOI (supplementary Table 1 and Figure 1)
  • Sensitivity analysis: Waning estimated by strain (supplementary Table 2)
  • Sensitivity analysis: Alpha and cut-off jointly simultaneously by study (supplementary Table 3 and Figure 2)
  • Reverse catalytic model (supplementary Figure 3 and Table 4)
  • Sensitivity analysis: Including the youngest age groups (<1 year) (supplementary Table 5 and Figure 4)
  • Sensitivity analysis: Refitting the model using data from only two strains (supplementary Table 6)
  • Sensitivity analysis: Waning estimated by assay (supplementary Table 7 and Figure 5)

Data are available under the terms of the Creative Commons Attribution 4.0 International license (CC-BY 4.0).

## Software availability

Source code available from: https://github.com/erees/seasonalHCoV

Archived source code at time of publication: https://doi.org/10.5281/zenodo.5707764 ^25^

License: GNU General Public License v3.0.
