# Supplementary material for: Estimating the duration of seropositivity of human seasonal coronaviruses using seroprevalence studies
Source: Wellcome Open Res. 2021 Dec 21;6:138. Originally published 2021 Jun 3. [Version 3] doi: 10.12688/wellcomeopenres.16701.3 (PMC8517721; doi:10.12688/wellcomeopenres.16701.3)
Supplement: Peer review history

# Peer review: Reviewer response for version 3

**DOI:** 10.21956/wellcomeopenres.19394.r47731

**Published:** 2022-01-04

**Authors:** Sereina A. Herzog

**Affiliations:** 1 Institute for Medical Informatics, Statistics and Documentation, Medical University of Graz, Graz, Austria

No comments on the current version 3.

Is the work clearly and accurately presented and does it cite the current literature?

Partly

If applicable, is the statistical analysis and its interpretation appropriate?

Yes

Are all the source data underlying the results available to ensure full reproducibility?

Yes

Is the study design appropriate and is the work technically sound?

Yes

Are the conclusions drawn adequately supported by the results?

Yes

Are sufficient details of methods and analysis provided to allow replication by others?

Yes

Reviewer Expertise:

Infectious disease modeling, biostatistics

I confirm that I have read this submission and believe that I have an appropriate level of expertise to confirm that it is of an acceptable scientific standard.

# Peer review: Reviewer response for version 2

**DOI:** 10.21956/wellcomeopenres.19266.r47156

**Published:** 2021-11-26

**Authors:** Angkana T. Huang

**Affiliations:** 1 Department of Biology and Emerging Pathogens Institute, University of Florida, Gainesville, FL, USA; 2 Armed Forces Research Institute of Medical Sciences, Bangkok, Thailand

I thank the authors for thoroughly considering and addressing my comments. I only have one minor point to make regarding my suggestion of ELPD but is not meant to hold off the state of the approval. Specifically, it would be great if the authors could also include the 95% CI (in addition to the point estimates of the performance metric) to demonstrate that the superiority was well supported (i.e., whether the intervals were non-overlapping).

Is the work clearly and accurately presented and does it cite the current literature?

Partly

If applicable, is the statistical analysis and its interpretation appropriate?

Yes

Are all the source data underlying the results available to ensure full reproducibility?

Yes

Is the study design appropriate and is the work technically sound?

Yes

Are the conclusions drawn adequately supported by the results?

Yes

Are sufficient details of methods and analysis provided to allow replication by others?

Yes

Reviewer Expertise:

infectious disease modeling

I confirm that I have read this submission and believe that I have an appropriate level of expertise to confirm that it is of an acceptable scientific standard.

# Response

**Published:** 2021-12-15

**Authors:** Eleanor Rees

**Affiliations:** London School of Hygiene & Tropical Medicine, UK

Thank you for taking the time to review the manuscript again.

 Thank you for the suggestion, we have now included the standard error estimates as a measure of the variance for both the WAIC and LOO. The standard errors for these point estimates are large, and we have highlighted this in the text.

# Peer review: Reviewer response for version 2

**DOI:** 10.21956/wellcomeopenres.19266.r47157

**Published:** 2021-11-23

**Authors:** Barnaby Flower

**Affiliations:** 1 Department of Infectious Disease, Faculty of Medicine, Imperial College London, London, UK; 2 Oxford University Clinical Research Unit, Vietnam, Ho Chi Minh City, Vietnam

No further comments.

Is the work clearly and accurately presented and does it cite the current literature?

Yes

If applicable, is the statistical analysis and its interpretation appropriate?

Yes

Are all the source data underlying the results available to ensure full reproducibility?

Yes

Is the study design appropriate and is the work technically sound?

Yes

Are the conclusions drawn adequately supported by the results?

Partly

Are sufficient details of methods and analysis provided to allow replication by others?

Yes

Reviewer Expertise:

SARSCoV2 antibody seroprevalence, SARSCoV2 diagnostics, Hepatitis C Clinical Trialist. No modeling expertise.

I confirm that I have read this submission and believe that I have an appropriate level of expertise to confirm that it is of an acceptable scientific standard.

# Peer review: Reviewer response for version 2

**DOI:** 10.21956/wellcomeopenres.19266.r47155

**Published:** 2021-11-22

**Authors:** Sereina A. Herzog

**Affiliations:** 1 Institute for Medical Informatics, Statistics and Documentation, Medical University of Graz, Graz, Austria

The authors have addressed the comments I had, and I have no further comments on the revised version.

Is the work clearly and accurately presented and does it cite the current literature?

Partly

If applicable, is the statistical analysis and its interpretation appropriate?

Yes

Are all the source data underlying the results available to ensure full reproducibility?

Yes

Is the study design appropriate and is the work technically sound?

Yes

Are the conclusions drawn adequately supported by the results?

Yes

Are sufficient details of methods and analysis provided to allow replication by others?

Yes

Reviewer Expertise:

Infectious disease modeling, biostatistics

I confirm that I have read this submission and believe that I have an appropriate level of expertise to confirm that it is of an acceptable scientific standard.

# Peer review: Reviewer response for version 1

**DOI:** 10.21956/wellcomeopenres.18416.r46084

**Published:** 2021-10-14

**Authors:** Sereina A. Herzog

**Affiliations:** 1 Institute for Medical Informatics, Statistics and Documentation, Medical University of Graz, Graz, Austria

Rees *et al. *provide estimations for the force of infection (FOI) and seropositivity waning rate regarding four different human coronaviruses (HCoV) strains using reverse catalytic model with age-varying FOI. The limitations of the modelling approach as well as the limitations about the datasets are well discussed.

 Some minor points that should be addressed:

1. The authors give in the introduction examples about pathogens which are ‘fully immunising’ like pertussis. To my knowledge pertussis natural infection protects longer than vaccination but not lifelong (see Wendelboe *et al.*, 2005 ^1^). Please rephrase the sentence accordingly.
2. Please clarify in the method section what is meant with “[…] were excluded, as well as studies with small number of data points.” Is ‘small number’ referring to number of different age (groups) reported in the study or number of total observations in a study, and what is ‘small’?
3. The full dataset used is not available on Zenodo (reference 25) but on GitHub (reference 33) [Method section, 1 ^st^ paragraph, last sentence] – please check throughout the text those two references (e.g. last sentence Method section GitHub is mentioned with reference number 25).
4. In the method section it is stated that for the age at the cut-off time (a0) also uniform priors from 0 to 5 years are also investigated, however, I don’t see this prior appearing in the Table 2 with the different models and priors or in the Result section.
5. Can the authors clarify in the discussion why they did not investigate a sensitivity analysis about the different assays used in the studies, e.g. allowing to vary waning according to assays used?
6. It is not clear which model was used to produce Figure 3B and which FOI estimates were used (from the main model, all models?) and how they were pooled. The description and interpretation of Figure 3B should be deepened.

Some minor points which could improve readability:

1. In the 3 ^rd^ paragraph of the introduction, the authors provide the number of observations for the studies mentioned in this paragraph except for the studies conducted in Kenya and in New York. It would help to put the results in perspective to also have the numbers for those two studies.
2. Assay description: Table 1 indicates a very rough description of the assay used in the study. For interested readers it would be helpful to have more information in the supplementary material about the assays like exact assay name, was it qualitative/quantitative, sensitivity/specificity, what cut-off values were used, what is the lower limit of detection (LLOD), what happened with equivocal results (count towards being positive or negative), etc.
3. Referring to tables and figures in the supplementary material: change ‘extended data Figure 1, Table 1’ to ‘Supplementary Figure 1, Table 1’ etc.
4. Table 1-4: add the abbreviations used within the table in the table legend (e.g. Table 1 HCoV).
5. Table 2 - 5 ^th^ model: maybe rephrase ‘using all data’ to ‘using also data on <1 year’.
6. Table 3: I would suggest to extend the column titles a bit: “FOI”: clarifying that it is the one for the younger age group (i.e. lambda1); “Cut-off”: clarifying that his is about splitting observations into two age groups and adding the unit years.
7. Table 4: Why are not all models from Table 2 reappearing in this table?

Is the work clearly and accurately presented and does it cite the current literature?

Partly

If applicable, is the statistical analysis and its interpretation appropriate?

Yes

Are all the source data underlying the results available to ensure full reproducibility?

Yes

Is the study design appropriate and is the work technically sound?

Yes

Are the conclusions drawn adequately supported by the results?

Yes

Are sufficient details of methods and analysis provided to allow replication by others?

Yes

Reviewer Expertise:

Infectious disease modeling, biostatistics

I confirm that I have read this submission and believe that I have an appropriate level of expertise to confirm that it is of an acceptable scientific standard, however I have significant reservations, as outlined above.

## References

[1] : Duration of immunity against pertussis after natural infection or vaccination. *Pediatr Infect Dis J* .2005;24(5 Suppl) : 10.1097/01.inf.0000160914.59160.41 S58-61 10.1097/01.inf.0000160914.59160.41 15876927

# Response

**Published:** 2021-11-17

**Authors:** Eleanor Rees

**Affiliations:** London School of Hygiene & Tropical Medicine, UK

*Thank you for taking the time to read our article. We have added an additional sensitivity analysis by assay and clarified and amended the technical points you raised. Please see our detailed responses to your comments below.*

 Some minor points that should be addressed:

1. The authors give in the introduction examples about pathogens which are ‘fully immunising’ like pertussis. To my knowledge pertussis natural infection protects longer than vaccination but not lifelong (see Wendelboe *et al.*, 2005 ^1^ ). Please rephrase the sentence accordingly.

*Thank you for bringing this to our attention, we have now corrected this.*

 2. Please clarify in the method section what is meant with “[…] were excluded, as well as studies with small number of data points.” Is ‘small number’ referring to number of different age (groups) reported in the study or number of total observations in a study, and what is ‘small’?

*We excluded Liang et al. 2013 since they only reported two age groups (ages 0 and 18-25). This has now been clarified in the text:*

*“Studies which did not include estimates for individuals under 10 years old were excluded, as well as studies with which only reported two age groups.”*

**

 3. The full dataset used is not available on Zenodo (reference 25) but on GitHub (reference 33) [Method section, 1 ^st^ paragraph, last sentence] – please check throughout the text those two references (e.g. last sentence Method section GitHub is mentioned with reference number 25).

*Thank you – these references have now been corrected throughout the text.*

 4. In the method section it is stated that for the age at the cut-off time (a0) also uniform priors from 0 to 5 years are also investigated, however, I don’t see this prior appearing in the Table 2 with the different models and priors or in the Result section.

*This is referred to as “cut-off” in all the tables and text. Uniform priors between 0 and 20 were chosen for the cut-off (a0), and these are stated in Table 2.  I have now clarified the description or the priors in the methods and added further information below the table to avoid confusion.*

*Methods:*

*“For the age at cut-off (a _0_), uniform priors from 0 to 20 years were chosen as we were interested in the difference in FOI in children and young adults.”*

 5. Can the authors clarify in the discussion why they did not investigate a sensitivity analysis about the different assays used in the studies, e.g. allowing to vary waning according to assays used?

*Following yours and the other reviewers suggestion we have now included an additional sensitivity in the supplementary material, where we estimated the duration of antibody persistence by assay. We found that when we allowed waning to vary by assay (ELISA, IFA, HI and Neutralisation) we estimated the duration of antibody persistence to be between 1.1 years to 2.6 years for ELISA, HI and neutralisation. For IFA we observed a longer estimate of 7.7 (CrI: 3.0-14.3) years. However, we found wide credible intervals, particularly for the neutralisation and IFA which only had one study setting per assay. This highlights the need for more studies looking at the seroprevalence of HCoVs, and for better standardisation of assays.*

*Results:*

*"Finally, we explored the impact of the different assays used in the studies on the waning estimates. We allowed the waning estimate to vary by assay (extended data Table 7, Figure 5 ^25 ^), whilst allowing FOI to vary by study, and alpha and cut-off to vary by setting. This model estimated the duration of antibody persistence to be similar for ELISA (2.63 [95% CrI: 0.94-9.09] years), HI (1.08 [95% CrI: 0.44-3.33] years) and neutralisation (1.28 [95% CrI: 0.25-50.0] years) assays, but longer for IFA (7.69 [95% CrI: 3.03-14.29] years). The credible intervals were wide, likely due to the small number of studies by assay."*

*Discussion:*

*"When we allowed the waning estimate to vary by assay, we found a similar estimates of antibody persistence for ELISA, HI and neutralisation assays, ranging from 1.1 years to 2.6 years, and these are comparable to the estimates from the main model. However, for IFA, we observed a longer estimate of 7.7 years (CrI: 3.0-14.3). Due to the small number of studies, the credible intervals were large, particularly for the IFA and neutralisation assay, which only had one study setting for each assay. This highlights the need for more studies, and better standardisation of assays."*

 6. It is not clear which model was used to produce Figure 3B and which FOI estimates were used (from the main model, all models?) and how they were pooled. The description and interpretation of Figure 3B should be deepened.

*Thank you for this comment. We have clarified within the figure legend which model was used for Fig 3B. We have also expanded the description and interpretation of the figure within the results section.*

*“To demonstrate the relationship between FOI and seropositivity at age 30, we created simulated scenarios under different sero-catalytic models. Using the parameters for the relative change in FOI and waning estimated from the age-varying reverse catalytic model (where the relative change in FOI and the age at cut-off were simultaneously estimated across settings), we simulated the proportion of individuals aged 30 years that would be seropositive using a range of FOI estimates to show how the proportion changes using the different models. The catalytic model, which does not allow for seroreversion, results in the highest estimates of seropositivity at age 30 with increasing FOI. The age-varying FOI model results in higher estimates of seropositivity at age 30 compared with the reverse catalytic model. This is due to the FOI which was estimated to be almost twice as high in the older age group (with age at cut-off 8.49 [7.52 – 9.94] years) in the age-varying FOI model ( Figure 3A). We further explored the relationship between FOI, attack rates and the estimated number of infections by age. We used the pooled estimate across all studies of FOI to estimate the proportion exposed at a given age to provide an indication of how many infections we might expect to see by age under our modelling assumptions ( Figure 3B). We estimate that by two years, over 50% of the population will have at least one infection, and by age ten over 75% will have had more than four infections.”*

 Some minor points which could improve readability:

1. In the 3 ^rd^ paragraph of the introduction, the authors provide the number of observations for the studies mentioned in this paragraph except for the studies conducted in Kenya and in New York. It would help to put the results in perspective to also have the numbers for those two studies.
*This has now been added to the introduction.*
2. Assay description: Table 1 indicates a very rough description of the assay used in the study. For interested readers it would be helpful to have more information in the supplementary material about the assays like exact assay name, was it qualitative/quantitative, sensitivity/specificity, what cut-off values were used, what is the lower limit of detection (LLOD), what happened with equivocal results (count towards being positive or negative), etc.
* Thank you, I have added some further information to Table 1 regarding the assay characteristics (antigen used and assay cut-off used in the study). I have also added an assay-specific sensitivity analysis and added further details regarding different assays in the discussion section. There is limited information in the literature on sensitivity and specificity of these assays, likely because these pathogens aren’t tested routinely in the community. Furthermore, given seroprevalence studies include asymptomatic and mildly symptomatic individuals, it is uncertain how these assays perform as validation is usually conducted in acute cases.*
3. Referring to tables and figures in the supplementary material: change ‘extended data Figure 1, Table 1’ to ‘Supplementary Figure 1, Table 1’ etc.

*Thank you for your suggestion, however, this is the naming convention chosen by Wellcome Open research.*

 4. Table 1-4: add the abbreviations used within the table in the table legend (e.g. Table 1 HCoV).

*These have been added.*

 5. Table 2 - 5 ^th^ model: maybe rephrase ‘using all data’ to ‘using also data on <1 year’.

*This has been amended.*

 6. Table 3: I would suggest to extend the column titles a bit: “FOI”: clarifying that it is the one for the younger age group (i.e. lambda1); “Cut-off”: clarifying that his is about splitting observations into two age groups and adding the unit years.

*Thank you for your suggestion, the table headings have been changed.*

 7. Table 4: Why are not all models from Table 2 reappearing in this table?

*Since we explored many different models, we chose to focus this table only on the models which we felt were the most relevant, particular since this table is a comparison summary table, and the detailed information for all models is provided elsewhere in the manuscript.*

# Peer review: Reviewer response for version 1

**DOI:** 10.21956/wellcomeopenres.18416.r46089

**Published:** 2021-10-12

**Authors:** Barnaby Flower

**Affiliations:** 1 Department of Infectious Disease, Faculty of Medicine, Imperial College London, London, UK; 2 Oxford University Clinical Research Unit, Vietnam, Ho Chi Minh City, Vietnam

Rees *et al.* provide a timely analysis of duration of natural immunity against seasonal human coronaviruses. They use an age-structured catalytic model which inputs historical seroprevalence data from five studies to estimate likely duration of seropositivity following seroconversion. They estimate HCoV antibodies last between 0.9 and 3.8 years, and find that the rate at which susceptible individuals acquire infection and seroconvert is higher in older children (>8.49 years) and adults than it is in younger children. The duration of seropositivity is consistent with previous estimates by Edridge *et al.* and tallies with emerging data concerning SARSCoV2.

 I am not a modeler and defer to my modeling colleague's review regarding aspects of the methodology. However to the non-specialist the methods appear robust and reproducible and they are transparently described in this well-written paper.

 As with all models, the outputs are only as good as the inputs, and in this case, data has been extracted from just 5 studies performed between 1965 and 2011 in four countries (Hong Kong, China, USA and Germany). The studies employ different assays, namely: enzyme-linked immunosorbent assays (ELISA), immunofluorescence assays (IFA), western blots, and complement fixation (CF), hemagglutination inhibition assays (HAI) and neutralisation assays which have different sensitivities and specificities, and I suspect this will lead to heterogeneous under-estimation of actual antibody positivity rates, particularly in comparison to current estimates of SARSCoV2 using highly sensitive ELISAs. The study would be strengthened by assay specific sensitivity analysis.

 The second limitation regards not accounting for seasonality. The authors point out that other studies have found that ignoring seasonality may overemphasize the role of adults in the transmission. Given that HCoVs are detected at much higher rates in the winter and spring seasons this makes it difficult to interpret one of the study's major findings of a higher force of infection in adults. This is mentioned in the 4th paragraph of the discussion but not in the context of the finding that the FOI was estimated to be twice as high in the older age group (6th paragraph) and perhaps this should be better tied together. 

 While these considerations might improve the paper, the limitations are well discussed and the manuscript is well worthy of indexing and dissemination. 

 One minor point:

 In paragraph 4 of the methods it says:

 "There is little information on the attack rate of HCoVs, but there have been several systematic reviews and meta-analyses looking at unvaccinated individuals which have reported the attack rate to range between 15.2% – 22.5% in children and 3.5% – 10.7% in adults ^26– 28^."

 This sentence is missing 'influenza'.

Is the work clearly and accurately presented and does it cite the current literature?

Yes

If applicable, is the statistical analysis and its interpretation appropriate?

Yes

Are all the source data underlying the results available to ensure full reproducibility?

Yes

Is the study design appropriate and is the work technically sound?

Yes

Are the conclusions drawn adequately supported by the results?

Partly

Are sufficient details of methods and analysis provided to allow replication by others?

Yes

Reviewer Expertise:

SARSCoV2 antibody seroprevalence, SARSCoV2 diagnostics, Hepatitis C Clinical Trialist. No modeling expertise.

I confirm that I have read this submission and believe that I have an appropriate level of expertise to confirm that it is of an acceptable scientific standard.

# Response

**Published:** 2021-11-17

**Authors:** Eleanor Rees

**Affiliations:** London School of Hygiene & Tropical Medicine, UK

*Thank you for your comments on this manuscript. As you suggest, we have added an additional sensitivity analysis by assay and expanded our discussion around seasonality. Please see our detailed responses to your comments below.*

 As with all models, the outputs are only as good as the inputs, and in this case, data has been extracted from just 5 studies performed between 1965 and 2011 in four countries (Hong Kong, China, USA and Germany). The studies employ different assays, namely: enzyme-linked immunosorbent assays (ELISA), immunofluorescence assays (IFA), western blots, and complement fixation (CF), hemagglutination inhibition assays (HAI) and neutralisation assays which have different sensitivities and specificities, and I suspect this will lead to heterogeneous under-estimation of actual antibody positivity rates, particularly in comparison to current estimates of SARSCoV2 using highly sensitive ELISAs. The study would be strengthened by assay specific sensitivity analysis.

*Thank you for this suggestion, we have added an additional sensitivity looking at how the estimates of waning antibody duration change by assay. We found that when we allowed waning to vary by assay (ELISA, IFA, HI and Neutralisation) we estimated the duration of antibody persistence to be between 1.1 years to 2.6 years for ELISA, HI and neutralisation. For IFA we observed a longer estimate of 7.7 (CrI: 3.0-14.3) years. However, we found wide credible intervals, particularly for the neutralisation and IFA which only had one study setting per assay. This highlights the need for more studies looking at the seroprevalence of HCoVs, and for better standardisation of assays.*

*Results:*

*"Finally, we explored the impact of the different assays used in the studies on the waning estimates. We allowed the waning estimate to vary by assay (extended data Table 7, Figure 5 ^25^), whilst allowing FOI to vary by study, and alpha and cut-off to vary by setting. This model estimated the duration of antibody persistence to be similar for ELISA (2.63 [95% CrI: 0.94-9.09] years), HI (1.08 [95% CrI: 0.44-3.33] years) and neutralisation (1.28 [95% CrI: 0.25-50.0] years) assays, but longer for IFA (7.69 [95% CrI: 3.03-14.29] years). The credible intervals were wide, likely due to the small number of studies by assay."*

*Discussion:*

*"When we allowed the waning estimate to vary by assay, we found a similar estimates of antibody persistence for ELISA, HI and neutralisation assays, ranging from 1.1 years to 2.6 years, and these are comparable to the estimates from the main model. However, for IFA, we observed a longer estimate of 7.7 years (CrI: 3.0-14.3). Due to the small number of studies, the credible intervals were large, particularly for the IFA and neutralisation assay, which only had one study setting for each assay. This highlights the need for more studies, and better standardisation of assays."*

 The second limitation regards not accounting for seasonality. The authors point out that other studies have found that ignoring seasonality may overemphasize the role of adults in the transmission. Given that HCoVs are detected at much higher rates in the winter and spring seasons this makes it difficult to interpret one of the study's major findings of a higher force of infection in adults. This is mentioned in the 4th paragraph of the discussion but not in the context of the finding that the FOI was estimated to be twice as high in the older age group (6th paragraph) and perhaps this should be better tied together. 

*Thank you for your comment. The seasonality of HCoVs is an important consideration, and we have now expanded this in the discussion:*

*“The time of year data collection occurred may influence our results, particularly given that the duration of antibody persistence is estimated to range between 0.9 (95% CrI: 0.6 - 1.6) years and 3.8 (95% CrI: 2.0 - 7.4) years. Data collection during high transmission periods would lead to an over estimate of both the FOI and the duration of antibody persistence. All the studies (except for Chan et al. ^23^ who did not report this information), included within this analysis collected data over at least a six-month period. For this reason, the timing of data collection and the seasonality is unlikely to have biased our results.”*

*We have also further considered the Ferrari et al. paper in the context of our results. Their paper looked at measles outbreaks in Niger, which have a very different epidemic profile, with erratic outbreaks that vary by orders of magnitude, and large periods between epidemics. The epidemic profile is different for seasonal coronaviruses and we think that regular cyclic outbreaks do not strongly bias the model, as found by Whitaker & Farrington. We have expanded this within the discussion:*

*“We also did not account for seasonality within this model, which may have under-estimated our FOI. Ferrari et al. ^42 ^found that ignoring seasonality may overemphasize the role of adults in the transmission, however, this was observed in measles in Niger, with outbreak peaks ranging over several orders of magnitude, and long periods between epidemics. The epidemic profile is different for seasonal coronaviruses, and therefore, this is unlikely to apply in this context.”*

 While these considerations might improve the paper, the limitations are well discussed and the manuscript is well worthy of indexing and dissemination. 

 One minor point:

 In paragraph 4 of the methods it says:

 "There is little information on the attack rate of HCoVs, but there have been several systematic reviews and meta-analyses looking at unvaccinated individuals which have reported the attack rate to range between 15.2% – 22.5% in children and 3.5% – 10.7% in adults ^26^ ^–^ ^28^ ."

 This sentence is missing 'influenza'.

*Thank you, this has now been corrected.*

# Peer review: Reviewer response for version 1

**DOI:** 10.21956/wellcomeopenres.18416.r44311

**Published:** 2021-06-28

**Authors:** Angkana T. Huang

**Affiliations:** 1 Department of Biology and Emerging Pathogens Institute, University of Florida, Gainesville, FL, USA; 2 Armed Forces Research Institute of Medical Sciences, Bangkok, Thailand

Leveraging data on other human coronaviruses (HCoV) that have been circulating for longer, the authors estimated a range of antibody waning rates that might apply to SARS-CoV-2. They performed multiple sensitivity analyses to assess the robustness of their estimates. Their results also pointed towards transmission being higher in older individuals.

 The authors provided ample background to understand the motivation and the approach of their work. Below are some minor points that I view should be addressed.

**1) **Please consider using the term ‘seropositivity waning rate’ instead of ‘immunity waning rate’ for ω as many of the assays in the dataset are binding assays which may not directly translate to protection, though correlated.

**2)** I appreciate that the authors included multiple sensitivity analyses in their study. I would like to suggest one more where waning rates are assay specific. The assays measure different functional aspects of the antibodies which may involve different subsets of the induced repertoires. Some hints exist in the authors’ results. Shao (2007) and Chan (2009) used IgG ELISA while Monto (1974) and Sarateanu (1980) were HI based. Looking at plots of the observed data, these tend to exhibit more prominent wanes. In Table S6, Model 1, 5, and 6 where Monto (1974) and Sarateanu (1980) were included, the estimated waning rates were much higher than the others. It could be that these were specific to HCoV-OC43, but looking at Table S2, the estimates for -NL63 and -HKU1 were similar to -OC43 (considering both the point estimates and credible intervals). Posterior density plots from code provided by the authors showed that combined cutoff of the Cavallaro (1970) and the Monto (1974) study in the main model, though converged, is bimodal. I wonder if this resulted from the model trying to accommodate the enforced single waning rate between studies which used different assays. This assessment will likely help the field reconcile discrepancies in serosurveys that were measured using different assays.

**3)** As the authors pointed out, data for some strains were from the same settings. This is evidence for co-circulation. The cross-reactivity between strains, specifically within alpha- and beta-CoVs, would affect the seropositivity. I acknowledge that literature is slim on what the degree of cross-reactivity is and the limited data may make it hard to infer from the data. Anyhow, please consider adding some discussion around how this would change the estimated waning rate as it will help readers adjust their expectations on what the true waning rates could be. One way to gain such intuition may involve extending the models to include cross-reactivity in studies with known co-circulation, imposing different sets of informative priors to the cross-reactivity rate, and assess how those affect the inferred waning rates. It is optional whether the authors would take on this path.

**4) **The authors may consider additional metrics of model performance, for instance, the expected log predictive density (ELPD) which is based on approximate leave-one-out cross-validation, https://doi.org/10.1007/s11222-016-9696-4. The measure provides uncertainties around the performance estimates which may help determine the superiority among the suite of models, especially for ones where DIC were very similar.

**5)** It would be helpful to provide an equation to explicitly show the link between the estimated waning rates and the duration of antibody persistence which were reported throughout the paper, and is the main finding of the study.

**6)** In contrast to the previous point, the authors provided an equation linking the FOI to the attack rates but barely touched upon this topic in the paper except for one occurrence in the Discussion. May there be ideas that the authors wished to discuss but were not fully expressed in the text?

**7)** It is unclear what the motivation for the analysis which led to Figure 3 was. Providing some context, especially how that would better our projections on SARS-CoV-2 would be helpful.

**8)** Lastly, there are some truncated sentences or missing/inconsistent information in the paper that needs to be fixed. For example:

- In the Methods, “The different assays used in each study for the different strains is shown, and where the antibody detected was specified this is included in the table.”
- In the Results, “When the relative change in FOI and cut-off parameters were simultaneously estimated by setting (extended data Figure 2, Table 3) the duration of antibody persistence was estimated to be shorter, …”.
- According to the captions, it occurs to me that the pairings are as follows:
  Table S1 with Figure S1, Table S3 with Figure S2, Table S5 with Figure S4. The pair of Table S2 is not obvious. It is unclear for Figure S3 which analysis this belongs to. Please also double check that the Extended data section in the main text matches the materials provided in the supplement.
- “The inference was impleented in RJags…”
- The figure legend of Figure 3A says “reverse catalytic & age varying FOI” while the caption says “reverse catalytic model with time-varying FOI”. The colors also do not seem to match the legend (blue vs green vs grey). May be helpful to choose colors that are more different, especially that the lines in the legend keys are quite thin.
- It is unclear which model was used to generate Figure 3B. I am guessing the “reverse catalytic model” from its matching color but explicit statements would be helpful to the readers.
- Please consider breaking this portion in the Discussion into a new sentence, “... and a meta-analysis of contact tracing studies found that children had 56% (31% – 71%) lower odds of becoming an infected contact compared with adults ^52^.”

Is the work clearly and accurately presented and does it cite the current literature?

Partly

If applicable, is the statistical analysis and its interpretation appropriate?

Yes

Are all the source data underlying the results available to ensure full reproducibility?

Yes

Is the study design appropriate and is the work technically sound?

Yes

Are the conclusions drawn adequately supported by the results?

Yes

Are sufficient details of methods and analysis provided to allow replication by others?

Yes

Reviewer Expertise:

infectious disease modeling

I confirm that I have read this submission and believe that I have an appropriate level of expertise to confirm that it is of an acceptable scientific standard, however I have significant reservations, as outlined above.

# Response

**Published:** 2021-11-17

**Authors:** Eleanor Rees

**Affiliations:** London School of Hygiene & Tropical Medicine, UK

Thank you for taking the time to review our article. We have added an additional sensitivity analysis by assay and technical details to the manuscript, as you suggest. Please see our detailed responses to your comments below.

**1) **Please consider using the term ‘seropositivity waning rate’ instead of ‘immunity waning rate’ for ω as many of the assays in the dataset are binding assays which may not directly translate to protection, though correlated.

*Thank you for this comment. We agree with the point you raise, and have amended “immunity waning rate” to “seropositivity waning rate” as suggested.*

**2)** I appreciate that the authors included multiple sensitivity analyses in their study. I would like to suggest one more where waning rates are assay specific. The assays measure different functional aspects of the antibodies which may involve different subsets of the induced repertoires. Some hints exist in the authors’ results. Shao (2007) and Chan (2009) used IgG ELISA while Monto (1974) and Sarateanu (1980) were HI based. Looking at plots of the observed data, these tend to exhibit more prominent wanes. In Table S6, Model 1, 5, and 6 where Monto (1974) and Sarateanu (1980) were included, the estimated waning rates were much higher than the others. It could be that these were specific to HCoV-OC43, but looking at Table S2, the estimates for -NL63 and -HKU1 were similar to -OC43 (considering both the point estimates and credible intervals). Posterior density plots from code provided by the authors showed that combined cutoff of the Cavallaro (1970) and the Monto (1974) study in the main model, though converged, is bimodal. I wonder if this resulted from the model trying to accommodate the enforced single waning rate between studies which used different assays. This assessment will likely help the field reconcile discrepancies in serosurveys that were measured using different assays.

*Thank you for this suggestion, we have added an additional sensitivity looking at how the estimates of waning antibody duration change by assay. We found that when we allowed waning to vary by assay (ELISA, IFA, HI and Neutralisation) we estimated the duration of antibody persistence to be between 1.1 years to 2.6 years for ELISA, HI and neutralisation. For IFA we observed a longer estimate of 7.7 (CrI: 3.0-14.3) years (See Extended data Figure 5 and Table 7). However, we found wide credible intervals, particularly for the neutralisation and IFA which only had one study setting per assay. This highlights the need for more studies looking at the seroprevalence of HCoVs, and for better standardisation of assays.*

*Results:*

*"Finally, we explored the impact of the different assays used in the studies on the waning estimates. We allowed the waning estimate to vary by assay (extended data Table 7, Figure 5 ^25^), whilst allowing FOI to vary by study, and alpha and cut-off to vary by setting. This model estimated the duration of antibody persistence to be similar for ELISA (2.63 [95% CrI: 0.94-9.09] years), HI (1.08 [95% CrI: 0.44-3.33] years) and neutralisation (1.28 [95% CrI: 0.25-50.0] years) assays, but longer for IFA (7.69 [95% CrI: 3.03-14.29] years). The credible intervals were wide, likely due to the small number of studies by assay."*

*Discussion:*

*"When we allowed the waning estimate to vary by assay, we found a similar estimates of antibody persistence for ELISA, HI and neutralisation assays, ranging from 1.1 years to 2.6 years, and these are comparable to the estimates from the main model. However, for IFA, we observed a longer estimate of 7.7 years (CrI: 3.0-14.3). Due to the small number of studies, the credible intervals were large, particularly for the IFA and neutralisation assay, which only had one study setting for each assay. This highlights the need for more studies, and better standardisation of assays."*

                        

**3)** As the authors pointed out, data for some strains were from the same settings. This is evidence for co-circulation. The cross-reactivity between strains, specifically within alpha- and beta-CoVs, would affect the seropositivity. I acknowledge that literature is slim on what the degree of cross-reactivity is and the limited data may make it hard to infer from the data. Anyhow, please consider adding some discussion around how this would change the estimated waning rate as it will help readers adjust their expectations on what the true waning rates could be. One way to gain such intuition may involve extending the models to include cross-reactivity in studies with known co-circulation, imposing different sets of informative priors to the cross-reactivity rate, and assess how those affect the inferred waning rates. It is optional whether the authors would take on this path.

*Thank you for this comment. We have added some additional discussion surrounding cross-reactivity and cross-protection to the discussion and explored how this might have impacted our results. Since we only have a limited number of datasets and data points, we don’t feel that we have sufficient data to include cross-reactivity within this analysis.*

*Discussion:*

*"In addition, we did not take into consideration cross-protection between seasonal coronavirus strains. There is some evidence of cross protective immunity between seasonal coronavirus strains, and in settings where there is co-circulating HCoV strains, this may lead to a higher prevalence. There is also evidence that there is cross-reactivity between different coronaviruses, which may lead to false positive results. A recent systematic review found that there was some cross-reactivity that occurred within alpha (HCoV-229E and HCoV-NL63) and beta (HCoV-OC43 and HCoV-HKU1) coronaviruses, but minimal reactivity between alpha and beta coronaviruses ^7 ^. However, it is not clear whether cross-reactivity equates to cross-protection. False positives due to cross-reactivity would lead to an over-estimation of seroprevalence in a setting. This would lead to a higher plateau in older ages, and therefore would generally lead to an over-estimation of both the FOI and the duration of antibody persistence."*

**4) **The authors may consider additional metrics of model performance, for instance, the     (ELPD) which is based on approximate leave-one-out cross-validation, https://doi.org/10.1007/s11222-016-9696-4. The measure provides uncertainties around the performance estimates which may help determine the superiority among the suite of models, especially for ones where DIC were very similar.

*Thank you for the suggestion. We agree that DIC as a metric has limitations for model selection, particularly given our DIC estimates were so similar, which is why in the main results and abstract we chose to report a range of estimates from the different models, rather than focussing simply on just one model. We have now added leave-one-out cross validation (LOO) to estimate the out of sample prediction ability. Additionally, we have added WAIC, which offers advantages over the DIC for Bayesian models, since it uses the entire posterior distribution. We have reported these for all models, and found the same general trends as observed using DIC.*

*“A sensitivity analysis was conducted using less informative priors for the FOI parameters, where a normal distribution was used (extended data Figure 1, Table 1 ^25 ^). This model estimated a shorter duration of antibody persistence [0.93 (95% CrI: 0.60 - 1.64) years]. The FOI across all studies and strains were higher, ranging from 0.09 (95% CrI: 0.04 - 0.16) to 3.22 (95% CrI: 1.95 - 4.85), with six studies reporting FOI estimates > one, which is equivalent to an attack rate of >63%. The relative change in FOI and cut-off were similar for both models. This model had a lower DIC (476.8 compared with 480.8; with a DIC difference of 4), WAIC (536.2 compared with 546.4), and LOO (546.9 compared with 560.9), which suggests that this model has an improved fit compared with the model with more informed priors. However, the high FOI estimates indicate that this model may be less plausible ( Table 4).”*

*“When the relative change in FOI and cut-off parameters were simultaneously estimated by setting (extended data Figure 2, Table 3 ^25 ^) the duration of antibody persistence was estimated to be shorter, 2.20 (95% CrI: 1.57 - 3.08) years, although the confidence intervals overlap with the main model. The FOI ranged from 0.04 (95% CrI: 0.03 - 0.06) to 0.88 (95% CrI: 0.67 - 1.19). The overall model DIC (548.2 compared with 480.8), WAIC (622.0 compared with 546.5) and LOO (633.2 compared with 560.9) were higher, indicating that this model did not have as much support (Table 4).”*

**5)** It would be helpful to provide an equation to explicitly show the link between the estimated waning rates and the duration of antibody persistence which were reported throughout the paper, and is the main finding of the study.

*The duration of antibody persistence was estimated a 1/seropositivity waning rate. I have now included this as an equation in the text.*

*“The duration of antibody persistence was estimated as follows:*

*Duration of antibody persistence = 1/ ω”*

**6)** In contrast to the previous point, the authors provided an equation linking the FOI to the attack rates but barely touched upon this topic in the paper except for one occurrence in the Discussion. May there be ideas that the authors wished to discuss but were not fully expressed in the text?

*Our primary motivation for exploring attack rates was to inform our priors for the FOI estimates using attack rates from influenza (reported on in the methods). We realise we could have been clearer, so we have clarified this in the methods. We also compare the attack rates estimated for seasonal HCoV with influenza in the results and discussion to ensure that model parameter estimates were within plausible ranges.*

*Methods:*

*“For the FOI, there is little information on the attack rate of HCoVs. However, there have been several systematic reviews and meta-analyses looking at influenza in unvaccinated individuals which have reported the attack rates to range between 15.2% – 22.5% in children and 3.5% – 10.7% in adults ^26– 28 ^. Modelling studies using serological influenza data predicted estimates from 20 – 60% ^29, 30 ^. Based on the epidemiology of these viruses in children ^31^, we expect the attack rate for HCoV may be lower. Therefore, we selected a Gamma distribution, with a mean of 0.3 (shape = 1.2 and scale = 0.25) and this corresponds to an attack rate of 26% and covers a range of plausible values. For the age at cut-off (a _0_), uniform priors from 0 to 20 years were chosen as we were interested in the difference in FOI in children and young adults.”*

*Results*:

*“A sensitivity analysis was conducted using less informative priors for the FOI parameters, where a normal distribution was used (extended data Figure 1, Table 1 ^25 ^). This model estimated a shorter duration of antibody persistence [0.93 (95% CrI: 0.60 - 1.64) years]. The FOI across all studies and strains were higher, ranging from 0.09 (95% CrI: 0.04 - 0.16) to 3.22 (95% CrI: 1.95 - 4.85), with six studies reporting FOI estimates > one, which is equivalent to an attack rate of >63%. The relative change in FOI and cut-off were similar for both models. This model had a lower DIC (476.8 compared with 480.8; with a DIC difference of 4), WAIC (536.2 compared with 546.4), and LOO (546.9 compared with 560.9), which suggests that this model has an improved fit compared with the model with more informed priors. However, the high FOI estimates indicate that this model may be less plausible ( Table 4).”*

*Discussion*:

*“When we used less informed priors for the FOI, a lower estimate of duration of seropositivity was obtained. However, this model produced higher estimates of FOI, with six studies reported FOI estimates in the young age group greater than one (attack rate >63%). There is limited information on the attack rate of seasonal HCoV, however there have been numerous studies looking at influenza. Previous systematic reviews have estimated the attack rate of influenza to be between 3.5% and 22.5% ^26– 28 ^, whilst modelling studies have estimated this to be higher, 20 – 60% ^29, 30 ^. Based on reporting rates of seasonal HCoV we would expect the attack rate to be lower than influenza. Therefore, this suggests that the results from the model with less informative priors are less plausible.”*

**7)** It is unclear what the motivation for the analysis which led to Figure 3 was. Providing some context, especially how that would better our projections on SARS-CoV-2 would be helpful.

*Thank you for this comment. Our motivation for this figure was to look at the relationship between FOI, attack rates, and the number of infections you might expect to observe in younger ages. As you suggest, we have expanded the interpretation of this figure in the last paragraph of the results:*

*“To demonstrate the relationship between FOI and seropositivity at age 30, we created simulated scenarios under different sero-catalytic models. Using the parameters for the relative change in FOI and waning estimated from the age-varying reverse catalytic model (where the relative change in FOI and the age at cut-off were simultaneously estimated across settings), we simulated the proportion of individuals aged 30 years that would be seropositive using a range of FOI estimates to show how the proportion changes using the different models. The catalytic model, which does not allow for seroreversion, results in the highest estimates of seropositivity at age 30 with increasing FOI. The age-varying FOI model results in higher estimates of seropositivity at age 30 compared with the reverse catalytic model. This is due to the FOI which was estimated to be almost twice as high in the older age group (with age at cut-off 8.49 [7.52 – 9.94] years) in the age-varying FOI model ( Figure 3A). We further explored the relationship between FOI, attack rates and the estimated number of infections by age. We used the pooled estimate across all studies of FOI to estimate the proportion exposed at a given age to provide an indication of how many infections we might expect to see by age under our modelling assumptions ( Figure 3B). We estimate that by two years, over 50% of the population will have at least one infection, and by age ten over 75% will have had more than four infections.”*

**8)** Lastly, there are some truncated sentences or missing/inconsistent information in the paper that needs to be fixed. For example:

*Thank you for highlighting these, these have all been fixed.*

- In the Methods, “The different assays used in each study for the different strains is shown, and where the antibody detected was specified this is included in the table.”

*This has been amended.*

  

- In the Results, “When the relative change in FOI and cut-off parameters were simultaneously estimated by setting (extended data Figure 2, Table 3) the duration of antibody persistence was estimated to be shorter, …”.

*This has been amended.*

  

- According to the captions, it occurs to me that the pairings are as follows:
  Table S1 with Figure S1, Table S3 with Figure S2, Table S5 with Figure S4. The pair of Table S2 is not obvious. It is unclear for Figure S3 which analysis this belongs to. Please also double check that the Extended data section in the main text matches the materials provided in the supplement.

*The contents of the extended data can be found at the end of the manuscript under the “extended data” section:*

**The supple*mentary contains the following: *

-
*•* *Sensitivity analysis: Less informed priors for FOI (supplementary Table 1 and Figure 1)*
*•* *Sensitivity analysis: Waning estimated by strain (supplementary Table 2)*
*•* *Sensitivity analysis: Alpha and cut-off jointly simultaneously by study (supplementary Table 3 and Figure 2)*
*•* *Reverse catalytic model (supplementary Figure 3 and Table 4)*
*•* *Sensitivity analysis: Including the youngest age groups (<1 year) (supplementary Table 5 and Figure 4)*
*•* *Sensitivity analysis: Refitting the model using data from only two strains (supplementary Table 6)*
*•* *Sensitivity analysis: Waning estimated by assay (supplementary Table 7 and Figure 5)*

*We have also included subheadings within the supplementary material to make it easier to follow.*

- “The inference was impleented in RJags…”

*This has been amended.*

- The figure legend of Figure 3A says “reverse catalytic & age varying FOI” while the caption says “reverse catalytic model with time-varying FOI”. The colors also do not seem to match the legend (blue vs green vs grey). May be helpful to choose colors that are more different, especially that the lines in the legend keys are quite thin.

*I have updated the figure caption to clarify the models used to create Figure 3. I have changed the colours to allow for a clearer distinction between the models.*

  

- It is unclear which model was used to generate Figure 3B. I am guessing the “reverse catalytic model” from its matching color but explicit statements would be helpful to the readers.

* I have updated the figure caption to clarify the model used to create Fig 3b.*

*“* *Figure 3.* *( **A**) Proportion of individuals age 30 who are seropositive for different estimates of force of infection (FOI). The catalytic model is shown in red, the reverse catalytic model in green, and the reverse catalytic model with age-varying FOI is shown in blue. Model estimates were used for the parameter values (relative change in FOI (alpha),1.93 [1.69 – 2.19]; waning, 0.45 [0.32 – 0.64]; cut-off, 8.49 [7.52 – 9.94]). ( **B**) Estimated proportion of individuals experiencing infections by age estimated from the age-varying reverse catalytic model (more informed priors) using the pooled median estimate across studies for FOI (0.46), and median estimates for waning (0.45), alpha (1.93) and cut-off (8.49).”*

- Please consider breaking this portion in the Discussion into a new sentence, “... and a meta-analysis of contact tracing studies found that children had 56% (31% – 71%) lower odds of becoming an infected contact compared with adults ^52^.”

*This has been amended.*
